# Case Report: Identification of SARS-CoV-2 in Cerebrospinal Fluid by Ultrahigh-Depth Sequencing in a Patient With Coronavirus Disease 2019 and Neurological Dysfunction

**DOI:** 10.3389/fmed.2021.629828

**Published:** 2021-02-22

**Authors:** Pan Xiang, Xinmin Xu, Xin Lu, Lili Gao, Huizhu Wang, Zhenpeng Li, Haofeng Xiong, Ruihong Li, Yanwen Xiong, Lin Pu, Tian Qin, Fangfang Jin, Hongyu Ren, Chuansheng Li, Jing Yang, Ming Zhang, Jie Gong, Xiaoping Chen, Han Zheng, Jianbo Tan, Yao Sun, Fei Zhao, Xuexin Hou, Yufeng Liu, Hebing Guo, Jingjing Hao, Biao Kan, Haijian Zhou, Yajie Wang, Jingyuan Liu

**Affiliations:** ^1^Department of Critical Care Medicine, Beijing Ditan Hospital, Capital Medical University, Beijing, China; ^2^Department of Clinical Laboratory, Beijing Ditan Hospital, Capital Medical University, Beijing, China; ^3^State Key Laboratory of Infectious Disease Prevention and Control, Chinese Center for Disease Control and Prevention, National Institute for Communicable Disease Control and Prevention, Beijing, China

**Keywords:** SARS-CoV-2, COVID-19, central nervous system, infection, metagenomic next-generation sequencing

## Abstract

We reported that the complete genome sequence of SARS-Coronavirus-2 (SARS-CoV-2) was obtained from a cerebrospinal fluid (CSF) sample by ultrahigh-depth sequencing. Fourteen days after onset, seizures, maxillofacial convulsions, intractable hiccups and a significant increase in intracranial pressure developed in an adult coronavirus disease 2019 patient. The complete genome sequence of SARS-CoV-2 obtained from the cerebrospinal fluid indicates that SARS-CoV-2 can invade the central nervous system. In future, along with nervous system assessment, the pathogen genome detection and other indicators are needed for studying possible nervous system infection of SARS-CoV-2.

## Introduction

Coronavirus disease 2019 (COVID-19) is a new illness that has become a pandemic. As of October 18th 2020, there have been more than 39.3 million confirmed cases and more than 1.1 million deaths worldwide. Severe acute respiratory syndrome-Coronavirus-2 (SARS-CoV-2), the causative pathogen of COVID-19, pre-dominantly affects the lungs and causes respiratory illness (1). However, this virus also infects the intestinal tract, urinary system, blood, and other organ systems (2). Furthermore, SARS-CoV-2 is also associated with meningitis/encephalitis (3). In a study of hospitalized COVID-19 patients in Wuhan, China, 36.4% of patients experienced neurological symptoms, including headache, anosmia, ageusia, confusion, seizure, and encephalopathy (4). A recent study reported that SARS-CoV-2 can productively infect human neural progenitor cells and brain organoids, highlighting the potential of direct viral involvement in neurological symptoms in COVID-19 patients (5). SARS-CoV-2 has been detected by real-time reverse transcription-polymerase chain reaction (RT-PCR) in COVID-19 cases with central nervous system (CNS) symptoms in Japan. However, no complete sequence of SARS-CoV-2 from cerebrospinal fluid (CSF) has been reported (6–8). Here, we used ultrahigh-depth metagenomic next generation sequencing (mNGS) to determine the complete genome sequence of SARS-CoV-2 from the CSF of a COVID-19 case with CNS symptoms. This provides evidence for the existence of SARS-CoV-2 in the CNS.

## Case Report

**Figure 1:**
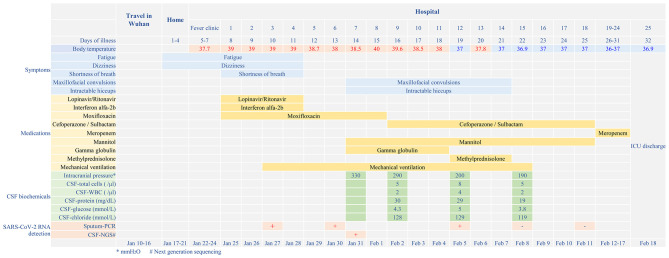
Disease course timeline from the initial presentation of illness to days spent in hospital.

**Figure 2:**
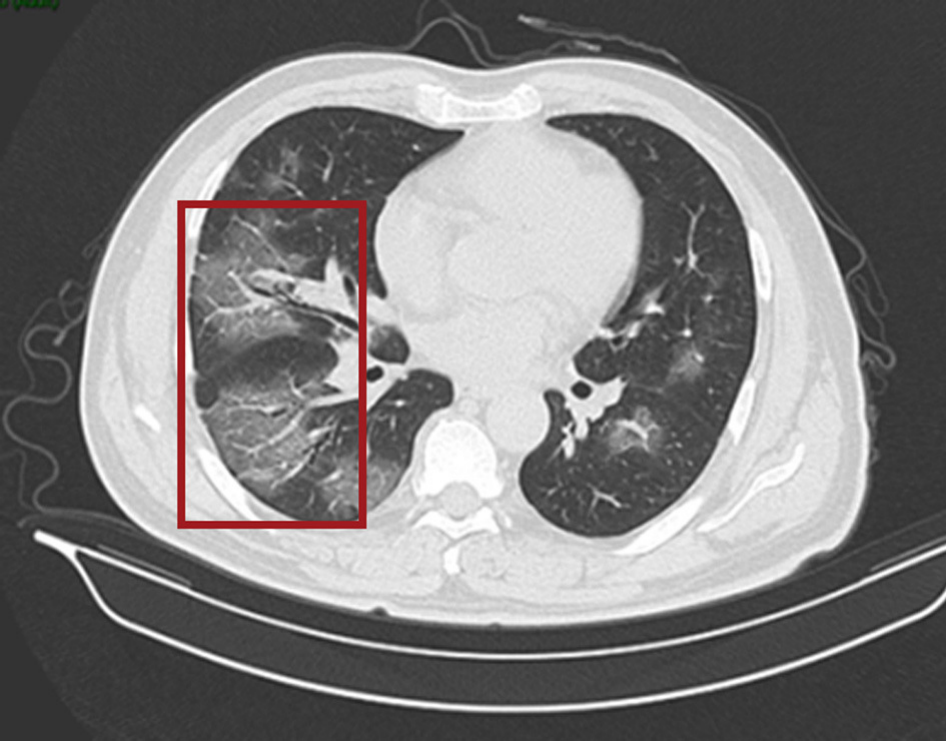
CT scan of the chest. Both lungs show scattered and patchy ground-glass opacities (in red box) on 24th January (day 7 of the illness).

On 24 January 2020, a 56-year-old man was admitted to hospital experiencing fatigue and dizziness for 7 days, and fever for 3 days (Figure 1). He had a history of hypertension in the preceding 3 years but his blood pressure was under normal control after treatment with amlodipine besylate. He had no lung disease, epilepsy, or familial psychosocial history. The patient had traveled to Wuhan 14 days prior to hospitalization and one of his relatives, who he had direct contact with, was a COVID-19 case. A computed tomography (CT) scan of the chest revealed a large area of ground-glass opacities (GGOs) dominated by extraneous areas in both lungs (Figure 2). A throat swab RT-PCR test for SARS-CoV-2 was positive. COVID-19 with respiratory failure then developed. A nasal catheter was inserted and oxygen was administered at 5 L/min. He was given antiviral therapy with lopinavir/ritonavir (500 mg twice daily) combined with interferon alfa-2b (5 million units twice daily, atomization inhalation), and moxifloxacin (0.4 g once daily, intravenously) to prevent secondary infection (Figure 1). After admission, his dyspnea symptoms continued to worsen. On the 10th day of illness, chest CT showed an enlarged GGO area and partial opacities in both lungs. Short-term high-flow nasal oxygen was briefly administered with a gas flow rate of 50 L/min and oxygen concentration of 90%. The patient continued to exhibit respiratory distress, with a respiratory rate of 50 times/min and oxygen saturation (SpO_2_) 85%. Therefore, endotracheal intubation was performed in the intensive care unit (ICU) and mechanical ventilation was conducted according to the respiratory ventilation protocol for severe acute respiratory distress syndrome.

**Figure 3:**
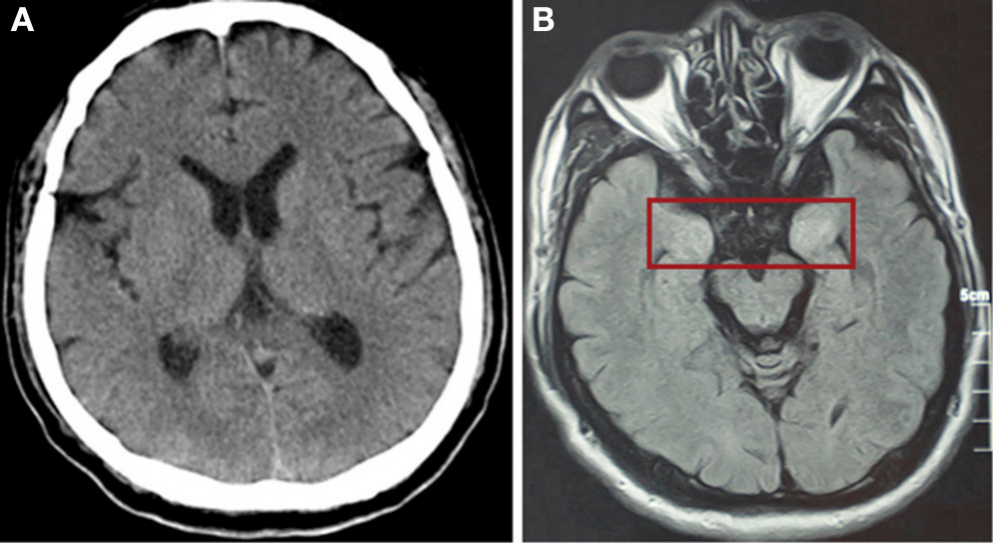
**(A)** CT scan of the brain showed no abnormally high or low opacity on 31st January (day 14 of the illness). **(B)** Magnetic resonance scan of the brain after discharge showed abnormalities (in red box) in the bilateral temporal lobe and hippocampus on 5th May (day 81 of the illness).

After mechanical ventilation for 96 h, frequent maxillofacial and oral spasms accompanied by persistent hiccoughs were observed. Physical examination revealed positive neck-resistance, bilateral pupils of equal size (3 mm diameter) with slow response to light, increased muscle tension in the extremities, hyperreflexia in both knees, and positive bilateral Babinski sign and ankle clonus. The CSF pressure was >330 mmH_2_O and had a clear colorless appearance. On the 14th day of illness, the patient was treated with gamma globulin (20 g daily for 5 days), mannitol dehydration (250 mL/6 h) to control intracranial pressure, chlorpromazine to control frequent hiccups, and midazolam (20 mg/h) to control seizures. Three days later, CSF pressure was >290 mmH_2_O, the CSF cell count was 5/mL, protein was 30 mg/mL and glucose was 4.3 mmol/L (Figure 1). No abnormalities were found on brain CT (Figure 3). Therefore, methylprednisolone (500 mg daily for 3 days) was given as shock therapy. Subsequently, the patient's hiccups disappeared and body temperature returned to normal. After discontinuation of midazolam on 6 February, the patient regained clear awareness of his surroundings and clinical seizures did not recur.

Meanwhile, pulmonary lesions were improved by mechanical ventilation for 14 days. The endotracheal intubation was removed on the 24th day of illness and the patient was discharged from ICU on the 32nd day of illness. Head magnetic resonance imaging on May 6 (day 82 of the illness) showed high signal shadows in the hippocampus and bilateral temporal lobe, which may indicate lesions (Figure 1). The patient had no cognitive or memory impairment after discharge.

### Metagenomic Sequencing

Total genomic DNA and RNA were extracted from samples using the QIAamp DNA Mini Kit and QIAamp Viral RNA Kit (Qiagen, USA), respectively, according to the manufacturer's protocols. Libraries were constructed with the MGIEasy FS DNA Library Prep Set, MGIEasy rRNA Depletion Kit, and MGIEasy RNA Library Prep Set (MGI, China). The prepared libraries were quantified using an Agilent 2100 (Agilent, USA) and sequenced on the MGISEQ-200RS and MGISEQ-2000RS sequencing platforms (MGI). NGS data were processed using Kraken (9), and the Pathogeny Fast Identification (PFI) pipeline for metagenomic identification of pathogens. Bowtie2 and samtools were used to extract the SARS-CoV-2 related reads. Available complete genomes of SARS-CoV-2 in the NCBI database were used as a reference. Based on the extracted SARS-CoV-2-related reads, we assembled the SARS-CoV-2 genome using SPAdes (10). To determine the Nextstrain clade of the sequence obtained in this study, 29 SARS-CoV-2 genome sequences belong to 19A/19B/20A/20B/20C/20D, according to the clade classification scheme of SARS-CoV-2 genomes by Nextstrain, were retrieved from GISAID (https://www.gisaid.org/). The genome sequences were aligned using mafft v7.45, and Mega v6.06 was used to infer the maximum likelihood tree.

**Figure 4:**
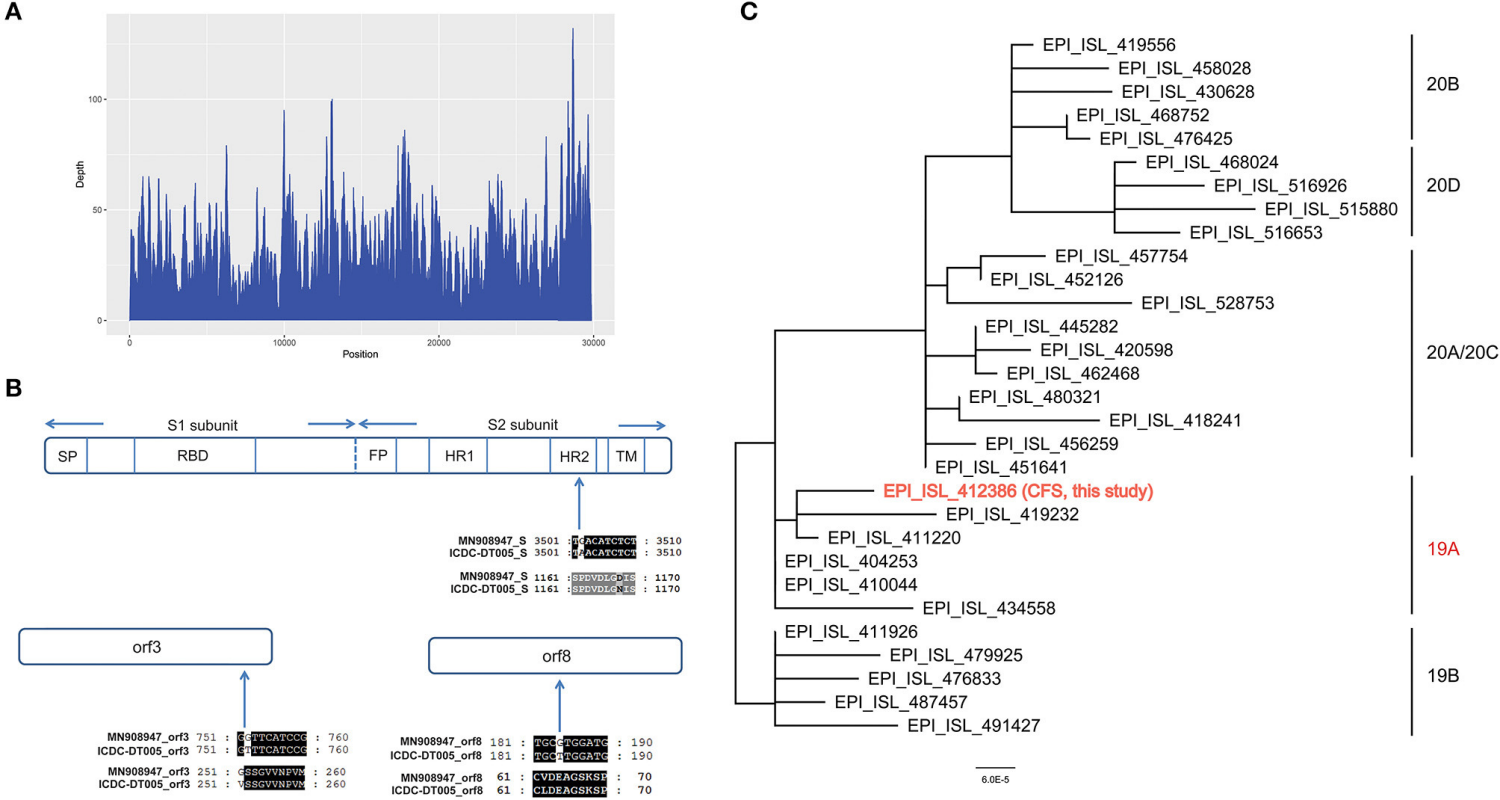
**(A)** Sequencing depth of the SARS-CoV-2 genome obtained in this study (EPI_ISL_412386). A total of 10,116 sequence reads derived from the patient's cerebrospinal fluid sample were mapped to the SARS-CoV-2 genome. **(B)** Alignment of the S gene, ORF3a and ORF8 of the SARS-CoV-2 genome obtained in this study (EPI_ISL_412386) with the reference genome of Wuhan-Hu-1. Only the alignment block containing a variation is shown for each protein. **(C)** Phylogenetic tree based on whole genome sequences of the SARS-CoV-2 genome obtained in this study (EPI_ISL_412386) and the 29 SARS-CoV-2 genomes which belong to 19A/19B/20A/20B/20C/20D obtained from GISAID.

Total genomic DNA and RNA were extracted from CSF and used to identify potential pathogens by RT-PCR assays and mNGS. The detection of SARS-CoV-2 by RT-PCR was negative, according to CT values >40. Ultrahigh-depth mNGS produced a full data set of 209,119,576 raw reads from the RNA library, and 10,116 reads corresponded to SARS-CoV-2. The reads were mapped to the SARS-CoV-2 reference genome NC_045512.2 and approximately 100% coverage was obtained with a mean depth of 31.6. No other pathogens were detected. We obtained a 29,857 bp SARS-CoV-2 genome (EPI_ISL_412386) (Figure 4) belonging to Nextstrain clade 19A. Furthermore, 29,003 and 15,790 bp SARS-CoV-2 genomes were assembled based on 61,224,674 and 8,800,232 raw reads from sputum and blood samples, respectively. No single nucleotide polymorphisms were found among the three assembled SARS-CoV-2 sequences from CSF, sputum and blood. Compared with the reference genome, MN908947, three nucleotide variations in EPI_ISL_412386 were found, all of which caused amino acid variations, including in the S2 subunit of the spike glycoprotein (D1168N), ORF3a (G251V), and ORF8 (V62L). No mutation was found in the receptor binding domain (Figure 4).

## Discussion

Human coronaviruses are recognized as respiratory viruses. However, some recognized human respiratory pathogens, including HCoV-OC43, HCoV-229E, and SARS-CoV, are associated with triggering or exacerbating neurological diseases because viral RNA or infectious virus can be detected in human brains (11–13). Preliminary reports showed some COVID-19 patients with CNS manifestations, such as dizziness, headache, nausea, vomiting, impaired consciousness, acute cerebrovascular disease, ataxia, and seizure, which indicated the neuroinvasive potential of SARS-CoV-2 (14, 15). Similar to SARS-CoV, SARS-CoV-2 also binds to the angiotensin-converting enzyme 2 (ACE2) receptor to enter human cells (16). Many types of cell in the brain, such as neurons and glial cells, express ACE2 and are thus vulnerable to SARS-CoV-2 infection. *In vitro* experiments show that SARS-CoV-2 can infect human neural progenitor cells and brain organoids (5). SARS-CoV-2 infection damages the choroid plexus epithelium, leading to leakage across this important barrier, which normally prevents entry of pathogens, immune cells, and cytokines into the CSF and the brain (17). Several case reports and post-mortem examinations of brain tissue have demonstrated SARS-CoV-2 nucleic acid in the CSF and brain tissue of infected and deceased individuals (18). Post-mortem of 43 COVID-19 patients showed fresh territorial ischemic lesions in 14% of patients, while 86% of patients had astrogliosis in all assessed regions. Activation of microglia and infiltration by cytotoxic T lymphocytes was most pronounced in the brainstem and cerebellum, and meningeal cytotoxic T lymphocyte infiltration was seen in 79% of patients (19). One case report patient, whose CSF was positive for SARS-CoV-2 based on RT-PCR, showed neurological symptoms of demyelinating disease (20). RT-PCR is currently the most popular method to detect SARS-CoV-2 because it is specific, rapid, and economical. However, SARS-CoV-2 infection is usually confirmed by monitoring one or two sites. Therefore, RT-PCR shows a high false negative rate in clinical assessments (21). Of 61 suspicious COVID-19 samples, 22 tested negative or inconclusive by RT-PCR but were identified as positive by sequencing (21). Therefore, sequencing has great potential for identifying viruses (22). Other methods can be used in parallel with mNGS. Intrathecal SARS-CoV-2 IgG and specific IgM in CSF have been found in RT-PCR assay-negative COVID-19 cases. These markers may be promising for the diagnosis of COVID-19 in cases with CNS symptoms (23). Calculation of the IgG antibody index (AI) is based on Reiber's method (24). In addition to analyzing the presence of viruses in CFS, its inflammatory profile, including white blood cell count and CSF/blood albumin ratio should also be determined as a supplement to mNGS (25). Among the COVID-19 patients in our care, the main neurological symptoms are maxillofacial convulsion, intractable burping, significantly increased intracranial pressure combined with neck resistance, positive bilateral Babinski sign and ankle clonus, which all indicate neurological dysfunction. There were no additional pathogens identified by examination of CSF. The whole genome sequence of SARS-CoV-2 was obtained from CSF by ultrahigh-depth mNGS, which revealed that SARS-CoV-2 had invaded the CNS. Not all neurological symptoms of COVID-19 occur because of direct viral action. Other causes can also lead to encephalopathy during viral infections, such as auto-immune encephalopathy; therefore, direct associations between encephalopathy symptoms and SARS-CoV-2 require further investigation. During the clinical treatment of this patient, autoimmune antibodies were not tested, and magnetic resonance imaging was not performed at the acute stage. Therefore, a causal relationship between the symptoms and the viral infection was not confirmed. We suggest that future studies include the detection of pleocytosis, high level of protein, blood-CSF barrier dysfunction, and intrathecal synthesis of immunoglobulins, which can be used to assess the inflammatory profile. Albuminocytological dissociation (high CSF protein levels without pleocytosis) can be assessed. Immunological and molecular examinations can be evaluated to exclude autoimmune encephalitis (23).

In summary, we present the first determination of the complete SARS-CoV-2 sequence based on ultrahigh-depth mNGS of a CSF sample, although a direct association between the symptoms of encephalopathy and SARS-CoV-2 requires further investigation. Our case confirmed that SARS-CoV-2 can invade the CNS, highlighting the need for physicians to pay close attention to nervous system symptoms of COVID-19 patients. Moreover, our results indicate that NGS can be used as a clinical approach for the diagnosis of a specific infectious disease caused by an uncommon pathogen. For COVID-19 patients with neurological dysfunction, detection of SARS-CoV-2 in CSF by NGS will provide a more comprehensive understanding of SARS-CoV-2 infection. This will help reduce the mortality of severely ill patients and lower the risk of transmission resulting from missed diagnosis.

## Data Availability Statement

The datasets presented in this study can be found in online repositories. The names of the repository/repositories and accession number(s) can be found at: GISAID, Accession ID: EPI_ISL_412386.

## Ethics Statement

The studies involving human participants were reviewed and approved by Beijing Ditan Hospital, Capital Medical University. The patients/participants provided their written informed consent to participate in this study.

## Author Contributions

BK, YW, and JL design the study. PX, XX, LG, HW, HX, RL, LP, FJ, CL, MZ, JT, YS, YL, HG, JH, YW, and JL supplied the clinical data. JL, PX, LG, HX, LP, CL, MZ, JT, YS, FZ, YL, HG, and JH evaluated and treated the patients. XL, YX, TQ, HR, JY, JG, XC, HZhe, FZ, XH, and HZho did the metagenomic next generation sequencing. HZho, PX, XX, XL, LG, ZL, and BK analyzed the data. HZho, PX, XL, and LG wrote the paper. BK, YW, and JL reviewed the paper. All authors contributed to the article and approved the submitted version.

## Conflict of Interest

The authors declare that the research was conducted in the absence of any commercial or financial relationships that could be construed as a potential conflict of interest.
